# Editorial: Innovative theranostic approaches towards neuro-immunology in gliomas

**DOI:** 10.3389/fimmu.2023.1122299

**Published:** 2023-02-21

**Authors:** Xiaoteng Cui, Lijie Zhai, Chunsheng Kang

**Affiliations:** ^1^ Laboratory of Neuro-oncology, Tianjin Neurological Institute, Tianjin Medical University General Hospital, Tianjin, China; ^2^ Department of Cancer Biology, Cardinal Bernardin Cancer Center, Loyola University Medical Center, Loyola University Chicago, Maywood, IL, United States

**Keywords:** immunotherapy, glioma, neuro-immunology, cancer, central nervous system

Since its announcement in 2013 in the journal ***Science*** as the breakthrough of the year (1), immunotherapy has essentially become the fourth pillar, in addition to surgery, radiation therapy and chemotherapy, in the current standard of care (SOC) treatment of many previously incurable cancers. Unfortunately, central nervous system (CNS)-derived malignant tumors have not fallen into this ever-growing list (2). The disappointing outcome of immunotherapy in CNS cancers, especially in gliomas, the most common CNS malignancy with a dismal prognosis, highlights the unique challenges in CNS tumor immunity in contrast to the immune regulation involved in peripheral tumors (3). This Research Topic was written with the aims of 1) introducing novel molecular targets, therapeutic approaches, diagnostic tools and techniques that have great potential in improving current glioma immunotherapy with regard to diagnosis/prognosis, treatment efficacy, response assessment, and immune correlative marker identification and 2) reviewing accumulating knowledge and the latest advancements in CNS tumor immunopathology to gain creative insight for the design of next-generation glioma immunotherapies.

Although multiple mechanisms contribute to the failure of current immunotherapy in the treatment of gliomas, the most prominent and notorious element is the strongly immunosuppressive tumor microenvironment (TME) associated with glioma tumorigenesis (4). A plethora of cellular and molecular components have been found to play important roles in glioma TME immunosuppression (5). Adding more to this list, several research studies in this collection investigated the correlations among newly identified molecules with the glioma TME and tumor infiltrating immune cells as well as immunotherapy responses. CD101, a transmembrane glycoprotein mainly expressed on dendritic cells, monocytes, and T cells, has been identified as a T-cell activation dampening molecule that is critical for T-cell immune tolerance. By analysis of the TCGA and GTE databases, Liu et al. demonstrated that intratumoral CD101 is an independent prognostic marker in glioma patients. High expression of CD101 is correlated with not only poor overall survival (OS) but also multiple immune regulatory signaling pathways. Interestingly, both bioinformatic and experimental data confirmed that immunosuppressive M2-like tumor-associated macrophages (TAMs) express high levels of CD101 in glioma patients. Lin et al. conducted a very similar study with a focus on mannosidase alpha class 2B member 1 (MAN2B1), a lysosomal alpha-d-mannosidase catalyzing the hydrolysis of mannoside linkages of N-linked glycoproteins. The same investigational approaches were utilized in several other studies that focused on various molecular markers, including the small nucleolar RNA host genes (SNHGs) by Fan et al., ubiquitin-specific protease 4 (USP4) by Tang et al., g-aminobutyric acidergic synapse-related genes (CSRGs) by Jiang et al., cyclin-dependent kinase regulatory subunit 2 (CKS2) by Yu et al., phosphatidylinositol binding clathrin assembly protein interacting mitotic regulator (PIMREG) by Zhu et al., and DNA damage response (DDR) by Chen et al. The identification of such a broad range of biomarkers as glioma prognosis predictors and their correlations with TME immune status as well as patients’ immunotherapy responses reiterate the extremely complex immune regulatory network involved in the glioma TME. It is of note that findings from these correlation studies, especially conclusions from bioinformatics analyses, still need further experimental validation. More importantly, the roles of these novel biomarkers in glioma tumorigenesis and immunopathology need to be confirmed functionally, and the underlying molecular and biochemical mechanisms remain largely unexplored.

Given that antiangiogenic therapy (bevacizumab) remains the top therapeutic option in the SOC treatment for the grimmest type of glioma, recurrent glioblastoma (6), it is timely and important that Lamplugh and Fan presented an elegant review on the impact of the vascular microenvironment on glioma immunity and immunotherapy. The corresponding molecular underpinnings and potentially contributing cellular components were thoroughly discussed with regard to vessel abnormality, microenvironment stress, immunosuppressive niche and adhesion dysfunction. Moreover, perspectives on reprogramming the vascular microenvironment by targeting vessel normalization and endothelial cell genetic engineering provide novel insights into future combination therapies to further improve glioma immunotherapy efficacy. In another closely related topic, Smith et al. provided a systemic review on the vascular endothelial growth factor (VEGF) coreceptor neuropilin-1 (NRP1) in high-grade gliomas. Their review article summarized the fundamental and diverse functionalities of NRP1 in endothelial cell biology, neuronal physiology and T lymphocytes, revealing a potential molecular target for future immune checkpoint blockade therapy in high-grade gliomas. Over the past decades, epigenetic modifications have been revealed to play vital roles in cancer immune modulation (7). A review article from Chen et al. introduced the basic concepts of N6-methyladenosine (m6A) RNA methylation and outlined the involvement of m6A methylation and the corresponding RNA methyltransferases in various cancer types. The immunomodulatory potential of m6A RNA methylation in gliomas was also discussed in a review of a series of correlational studies.

As cell-based therapies are a major part of current glioma immunotherapy, we are glad to include a comprehensive review article from Wang and Wang on the topic of advanced cell therapies for gliomas, which provided a well-organized overview of the latest advancements in chimeric antigen receptor T cells (CAT-T), natural killer (NK) cells and CAR-NK cells, gamma delta T cells, natural killer T (NKT) cells, and monocytes/dendritic/macrophages, as well as stem cell-based therapies. Adding more to this Research Topic, Tang et al. provided an overview of the advances in nanotechnology-based immunotherapy for glioblastoma (GBM), with an emphasis on the application of novel nanoparticle techniques to enhance a variety of glioma immunotherapeutic approaches.

Finally, two review articles focused on noninvasive diagnostic technologies for gliomas. Wadden et al. reviewed the latest advances in cell-free tumor DNA (cf-tDNA) liquid biopsy in gliomas. They summarized the current and future trends of various approaches for cf-tDNA detection and analysis with an emphasis on how to overcome the challenges of low sensitivity encountered in current cf-tDNA detection. Insights were also provided on the applications of cf-tDNA in different aspects of glioma immunotherapy. As the stellar technology for glioma noninvasive diagnosis, radiological imaging is essential for every aspect related to glioma patient care. In this article collection, Zhu et al. presented a comprehensive review on artificial intelligence (machine learning) technology in radiological imaging-based radiomic analysis in GBM. Written in a manner that is very friendly to noncomputer science background readers, this article introduced basic concepts and procedures of AI-assisted radiomic analysis and the associated problems and challenges. It also highlighted current applications of AI technology in various GBM clinical management needs, including differential diagnosis of GBM and classification, OS prediction, biomarker identification, and tumor immune response assessment.

This Research Topic is well balanced with manuscripts featuring a broad range of research interests that converge on the topic of neuro-immunology of gliomas. There are still many critical aspects that have not been covered in this collection and are worth further pursuing in the future, such as pediatric brain tumors, which possess significantly different immune landscapes in comparison to adult gliomas, aging-related immunosenescence, and key differences between CNS immune systems versus peripheral immune systems with regard to brain tumor immunity.

## Author contributions

All authors listed have made substantial, direct, and intellectual contributions to the work and approved it for publication.
